# Evaluation of the Immunomodulatory Ability of Lactic Acid Bacteria Isolated from Feedlot Cattle Against Mastitis Using a Bovine Mammary Epithelial Cells In Vitro Assay

**DOI:** 10.3390/pathogens9050410

**Published:** 2020-05-25

**Authors:** Kohtaro Fukuyama, Md. Aminul Islam, Michihiro Takagi, Wakako Ikeda-Ohtsubo, Shoichiro Kurata, Hisashi Aso, Graciela Vignolo, Julio Villena, Haruki Kitazawa

**Affiliations:** 1 Food and Feed Immunology Group, Laboratory of Animal Products Chemistry, Graduate School of Agricultural Science, Tohoku University, Sendai 980-8572, Japan; fukuyama.k.mc0511@gmail.com (K.F.); aminul.vmed@bau.edu.bd (M.A.I.); takagimichihiro@gmail.com (M.T.); wakako.ohtsubo.a7@tohoku.ac.jp (W.I.-O.); 2 Livestock Immunology Unit, International Education and Research Centre for Food and Agricultural Immunology (CFAI), Graduate School of Agricultural Science, Tohoku University, Sendai 980-8572, Japan; asosan@tohoku.ac.jp; 3 Department of Medicine, Faculty of Veterinary Science, Bangladesh Agricultural University, Mymensingh-2202, Bangladesh; 4 Laboratory of Molecular Genetics, Graduate School of Pharmaceutical Sciences, Tohoku University, Sendai 980-8572, Japan; shoichiro.kurata.d5@tohoku.ac.jp; 5 Cell Biology Laboratory, Graduate School of Agricultural Science, Tohoku University, Sendai 980-8572, Japan; 6 Reference Laboratory for Lactobacilli, (CERELA-CONICET), Tucuman-4000, Argentina

**Keywords:** mastitis control, immunobiotics, bovine mammary epithelial cells, innate immunity

## Abstract

Bovine mastitis, the inflammation of the mammary gland, affects the quality and quantity of milk yield. Mastitis control relies on single or multiple combinations of antibiotic therapy. Due to increasing antibiotic resistance in pathogens, the intramammary infusion of lactic acid bacteria (LAB) has been considered as a potential alternative to antibiotics for treating and preventing bovine mastitis through the improvement of the host immunity. Probiotic effects are a strain-dependent characteristic; therefore, candidate LAB strains have to be evaluated efficiently to find out the ones with the best potential. Here, we investigated LAB strains originally isolated from feedlot cattle’s environment regarding their ability in inducing the Toll-like receptor (TLR)-triggered inflammatory responses in bovine mammary epithelial (BME) cells in vitro. The BME cells were pre-stimulated with the LAB strains individually for 12, 24, and 48 h and then challenged with *Escherichia coli*-derived lipopolysaccharide (LPS) for 12 h. The mRNA expression of selected immune genes—interleukin 1 alpha (*IL-1α*), *IL-1β*, monocyte chemotactic protein 1 (*MCP-1*), *IL-8*, chemokine (C-X-C motif) ligand 2 (*CXCL2*), and *CXCL3* were quantified by real-time quantitative PCR (RT-qPCR). Results indicated that pretreatment with some *Lactobacillus* strains were able to differentially regulate the LPS inflammatory response in BME cells; however, strain-dependent differences were found. The most remarkable effects were found for *Lactobacillus acidophilus* CRL2074, which reduced the expression of *IL-1α*, *IL-1β*, *MCP-1*, *IL-8*, and *CXCL3*, whereas *Lactobacillus rhamnosus* CRL2084 diminished *IL-1β*, *MCP-1*, and *IL-8* expression. The pre-stimulation of BME cells with the CRL2074 strain resulted in the upregulated expression of three negative regulators of the TLRs, including the ubiquitin-editing enzyme A20 (also called tumor necrosis factor alpha-induced protein 3, TNFAIP3), single immunoglobin IL-1 single receptor (SIGIRR), and Toll interacting protein (Tollip) after the LPS challenge. The CRL2084 pre-stimulation upregulated only Tollip expression. Our results demonstrated that the *L. acidophilus* CRL2074 strain possess remarkable immunomodulatory abilities against LPS-induced inflammation in BME cells. This *Lactobacillus* strain could be used as candidate for in vivo testing due to its beneficial effects in bovine mastitis through intramammary infusion. Our findings also suggest that the BME cells immunoassay system could be of value for the in vitro evaluation of the immunomodulatory abilities of LAB against the inflammation resulting from the intramammary infection with mastitis-related pathogens.

## 1. Introduction

Bovine mastitis is defined as the inflammation of the mammary gland, which greatly affects milk production, animal health and welfare, and economic profit of dairy worldwide [1]. Mastitis is caused due to infection by several of Gram-positive and Gram-negative bacteria, resulting in a varying degree of clinical signs ranging from asymptomatic sub-clinical infection to severe acute mammary gland inflammation [2,3]. Studies have shown that intramammary infection of *Escherichia coli* resulted in severe clinical illness that is characterized by an acute inflammation through the vigorous stimulation of cytokine and chemokine synthesis [4,5].

Due to the multiple bacterial etiology, the treatment regime for clinical mastitis mostly relies on antibiotic therapy to minimize the morbidity [6]. Prophylactic intramammary infusion of long-acting antibiotics is frequently practiced to prevent intramammary infection in a dry period known as “dry cow therapy” [7]. For both prophylactic and therapeutic cases, a single or a combination of multiple antibiotics can be prescribed. However, cure rate of mastitis depends on the species of mastitis-causing pathogens, the efficacy of antibiotics, as well as the host immune status [6,8]. It has been well documented that irrational antibiotic therapy often leads to the development of antimicrobial resistance that poses a severe threat to food animal health and production. Resistance to bovine mastitis can also cause significant public health hazards though the transmission of antibiotic-resistant bacterial pathogens as well as antibiotic residues through the consumption of raw milk of antibiotic-treated cows [9]. Because of the increased probability of transmission of antibiotic resistance genes to indigenous and potential pathogens through antibiotic therapy as well as the poor cure rates of mastitis during lactation [10,11], the conventional treatment method needs to be revisited, and innovative and sustainable therapeutic alternatives should be sought.

Probiotics, which are considered as generally recognized as safe (GRAS) microorganisms, are defined as “live microorganisms which when administered in adequate amounts confer a physiological health benefit on the host” [12]. Among probiotics, those that exert their beneficial effects through the modulation of the host immune system are termed as “immunobiotics” [13]. Several lactic acid bacteria (LAB) have probiotic/immunobiotic properties, although this is a strain-dependent characteristic. For the identification and selection of beneficial LAB strains that can be used as probiotics, there are some criteria recommended by international organizations [12]. For example, probiotics are generally host-specific and believed to be more effective in their natural habitat [14]. In addition, the beneficial effects of probiotics/immunobiotics should be scientifically demonstrated in the host or a host-related system towards which the probiotic is directed.

It has been reported that LAB located on teat epithelia, in bedding materials, or in milk are able exert probiotic effects [15,16]. Then, the intramammary infusion of probiotics has been proposed as one of the most promising alternatives for the prevention and control of bovine mastitis [17,18,19,20,21,22,23]. The adhesion to epithelial cells and colonization of the mucosal tissue, the competition for nutrients, as well as the production of antimicrobial compounds are major pathogen-inhibitory mechanisms of LAB when administered into the bovine mammary gland [17]. In addition, the modulation of host immune response, in particular the capacity to differentially modulate the Toll-like receptor (TLR)-mediated innate immunity in mammary epithelia cells, is considered as one important characteristic of immunobiotic strains against mastitis [17]. 

Considering this background, the aim of this study was to select and characterize potential immunobiotic LAB strains that could be effectively used in the prevention or treatment of bovine mastitis. For this purpose, we took advantage of two scientific advances recently achieved by our research groups. On the one hand, we developed an immortalized bovine mammary epithelial (BME) cell line [24] and characterized it in terms of its ability to serve as a valuable in vitro tool for the study of the host–microbial interaction and the modulation of the mammary epithelial innate immunity triggered in response to infections. Our recent transcriptomic studies using the BME cells in vitro system have identified some potential immunological biomarkers for the evaluation of immunomodulatory probiotic candidates for the prevention or control bovine mastitis [25]. On the other hand, several LAB strains were isolated from the feedlot cattle environment [26] and characterized in terms of their potential probiotic properties. Among them, *Lactobacillus mucosae* CRL2069, *Lactobacillus acidophilus* CRL2074, *Lactobacillus fermentum* CRL2085, and *Lactobacillus rhamnosus* CRL2084 were reported to be free of transmissible antibiotic resistance genes and virulence factors, and capable of reaching a high number in in vitro culture media [27], indicating their optimal immunological potentials. Moreover, in a recent study, the ability of lactobacilli isolated from a feedlot cattle environment to differentially modulate the innate immune response triggered by TLR activation in bovine intestinal epithelial (BIE) cells was evaluated. Our results demonstrated that *L. mucosae* CRL2069 and *L. rhamnosus* CRL2084 had a remarkable capacity to modulate TLR4-mediated inflammation in BIE cells through the up-regulation of TLR-negative regulators, which in turn modulate the intracellular signaling pathways and reduce the expression of pro-inflammatory cytokines and chemokines [28]. We therefore aimed herein to further evaluate the ability of feedlot cattle lactobacilli to modulate the bovine mammary gland innate immunity triggered by *Escherichia coli* (*E. coli)*-derived lipopolysaccharide (LPS) using the BME cells in vitro system. 

## 2. Results

### 2.1. Expression Dynamics of TLR2 and TLR4 in BME Cells after Ligand Stimulation

**Figure 1:**
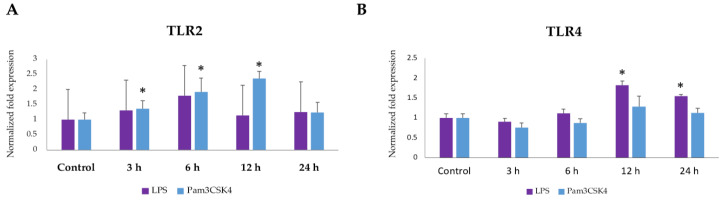
Expression dynamics of Toll-like receptor 2 (*TLR2*) and Toll-like receptor 4 (*TLR4*) in bovine mammary epithelial (BME) cells after ligand stimulation. BME cells (2.5 × 10^5^ cells/well) were stimulated with lipopolysaccharide (LPS) (1.0 μg/mL) or Pam3CSK4 (200 ng/mL) for 3, 6, 12, and 24 h. Then, mRNA expression of TLR2 (**A**) and TLR4 (**B**) were measured by RT-qPCR. Results presented are as mean ± SD of three independent experiments. * *p* < 0.05 indicates significant difference against control.

The development of innate immune responses against Gram-positive and Gram-negative bacterial infections is launched by the activation of TLR2 and TLR4, respectively. Thus, we first aimed to evaluate the expressions of *TLR2* and *TLR4* in BME cells in response to the ligands Pam3CSK4 and LPS. The Pam3CSK4 stimulation resulted in significant upregulation of *TLR2* expression in BME cells at 3 h, 6 h and 12 h post-stimulation (Figure 1). There was an increasing trend of *TLR2* expression in BME cells after LPS stimulation, but it was not statistically significant (Figure 1). Significant upregulation of *TLR4* expression was observed in BME cells at 12 and 24 h after stimulation with LPS. On the contrary, *TLR4* expression was not altered after the stimulation of BME cells with Pam3CSK4 (Figure 1).

### 2.2. Modulation of LPS-Induced Cytokine Expression in BME Cells by Lactobacilli 

**Figure 2:**
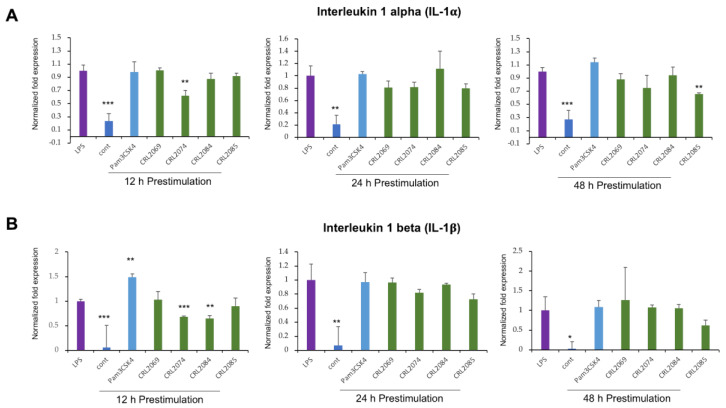
Evaluation of the immunomodulatory activity of feedlot cattle lactobacilli in bovine mammary epithelial (BME) cells. BME cells (2.5 × 10^5^ cells/well) were pre-stimulated either with *Lactobacillus mucosae* CRL2069, or *Lactobacillus acidophilus* CRL2074, or *Lactobacillus rhamnosus* CRL2084, or *Lactobacillus fermentum* CRL2085, or Pam3CSK4 for 12, 24, or 48 h, followed by a challenge with lipopolysaccharides (LPS) for further 12 h. Then, mRNA expression of interleukin 1 alpha (*IL-1α*) (**A**) and IL-1*β* (**B**) were measured by RT-qPCR. Results presented are as mean ± SD of three independent experiments. LPS, LPS-challenged cells without lactic acid bacteria (LAB) pre-stimulation; cont, untreated control cells; * *p* < 0.05, ** *p* < 0.01, *** *p* < 0.001 are the significant differences against LPS.

Taking into consideration that the duration of bacterial pre-stimulation is a determining factor for the epithelial cellular response, we performed experiments with three time points to evaluate the temporal effect of LAB pretreatment in preventing subsequent inflammatory challenge. For this purpose, BME cells were pre-stimulated with *L. mucosae* CRL2069, *L. acidophilus* CRL2074, *L. rhamnosus* CRL2084, *L. fermentum* CRL2085, or Pam3CSK4 for 12, 24, or 48 h, followed by a challenge with LPS for further 12 h. Then, mRNA expression of two proinflammatory cytokines—interleukin 1 alpha (*IL-1α*) and *IL-1β*—were measured by RT-qPCR (Figure 2). Results indicated that LPS challenge resulted in a significant augmentation of *IL-1α* expression in BME cells as compared to untreated control cells. The pre-stimulation of BME cells with *L. acidophilus* CRL2074 for 12 h and *L. fermentum* CRL2085 for 48 h was able to reduce the LPS-induced expression of *IL-1α* (Figure 2A). The pre-stimulation of Pam3CSK4 resulted in an upward trend of LPS-induced expression of IL-1*α* in BME cells (Figure 2A). Similar to *IL-1α* expression, the LPS challenge resulted in a significant increase of the expression of *IL-1β* in BME cells. *L. acidophilus* CRL2074 and *L. rhamnosus* CRL2084 showed significant ability to reduce the expression of *IL-1β* in LPS-challenged BME cells with 12 h of pre-stimulation (Figure 2B). In addition, the pre-stimulation with Pam3CSK4 resulted in upregulation of LPS-induced expression of *IL-1β* in BME cells (Figure 2B).

### 2.3. Modulation of LPS-Induced Nutrophil Chemoattractants in BME Cells by Lactobacilli

**Figure 3:**
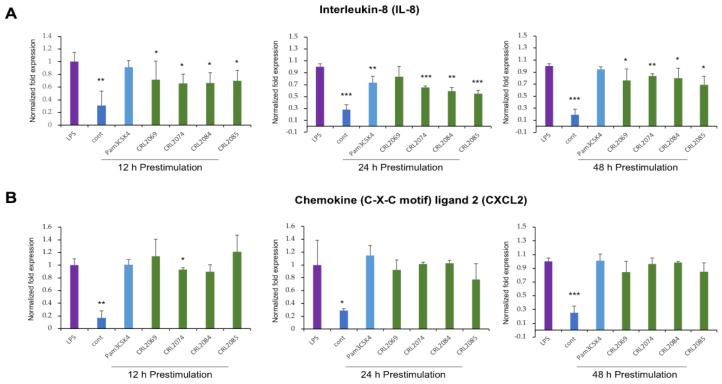
Evaluation of the immunomodulatory activity of lactobacilli in bovine mammary epithelial (BME) cells. BME cells (2.5 × 10^5^ cells/well) were pre-stimulated either with *L. mucosae* CRL2069, or *L. acidophilus* CRL2074, or *L. rhamnosus* CRL2084, or *L. fermentum* CRL2085, or Pam3CSK4 for 12, 24, or 48 h, followed by a challenge with lipopolysaccharides (LPS) for further 12 h. Then, mRNA expression of interleukin 8 (IL-8) (**A**) and chemokine (C-X-C motif) ligand 2 (CXCL2) (**B**) were measured by RT-qPCR. Results presented are as mean ± SD of three independent experiments. LPS, LPS-challenged cells without LAB pre-stimulation; cont, untreated control cells; * *p* < 0.05, ** *p* < 0.01, *** *p* < 0.001 are the significant differences against LPS.

In order to evaluate the ability of the feedlot cattle *Lactobacillus* strains to modulate the chemotaxis of immune cells into the infected mammary gland, we estimated the expression dynamics of two selected neutrophil chemoattractants in BME cells. The BME cells were pre-stimulated with *L. mucosae* CRL2069, *L. acidophilus* CRL2074, *L. rhamnosus* CRL2084, *L. fermentum* CRL2085, or Pam3CSK4, followed by a challenge with LPS (Figure 3). 

Interleukin 8 (IL-8), also known as chemokine (C-X-C motif) ligand 8 (CXCL8), is a chemoattractant and neutrophil activator. All the feedlot cattle lactobacilli evaluated here were able to significantly reduce the LPS challenge-induced expression of *IL-8* in BME cells (Figure 3A). The pre-stimulation of BME cells for 24 h with Pam3CSK4 also reduced the LPS-induced *IL-8* expression in BME cells (Figure 3A).

The chemokine (C-X-C motif) ligand 2 (CXCL2), also called macrophage inflammatory protein 2 alpha (MIP-2*α*), is a protein chemotactic factor for neutrophils. As shown in Figure 3B, only the pre-stimulation with *L. acidophilus* CRL2074 for 12 h was able to significantly reduce the expression of CXCL2 in LPS-challenged BME cells. 

### 2.4. Modulation of LPS-Induced Monocyte Chemoattractants in BME Cells by Lactobacilli

**Figure 4:**
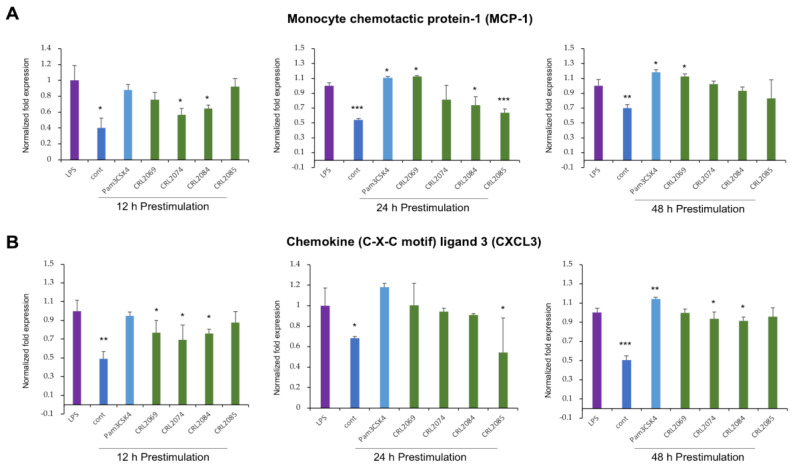
Evaluation of the immunomodulatory activity of lactobacilli in bovine mammary epithelial (BME) cells. BME cells (2.5 × 10^5^ cells/well) were pre-stimulated either with *L. mucosae* CRL2069, or *L. acidophilus* CRL2074, or *L. rhamnosus* CRL2084, or *L. fermentum* CRL2085, or Pam3CSK4 for 12, 24, or 48 h, followed by a challenge with lipopolysaccharides (LPS) for further 12 h. Then, mRNA expression of monocyte chemotactic protein 1 (MCP-1) (**A**) and chemokine (C-X-C motif) ligand 3 (*CXCL3*) (**B**) were measured by RT-qPCR. Results presented are as mean ± SD of three independent experiments. LPS, LPS-challenged cells without LAB pre-stimulation; cont, untreated control cells; * *p* < 0.05, ** *p* < 0.01, *** *p* < 0.001 are the significant differences against LPS.

The monocyte chemotactic protein 1 (MCP-1), also known as chemokine (C-C motif) ligand 2 (CCL2), is one of the most important chemokines that controls the migration and infiltration of monocytes and macrophages into the infected tissues. The pre-stimulation of BME cells with *L. acidophilus* CRL2074 for 12 h or *L. rhamnosus* CRL2084 for 12 or 24 h minimized the LPS-induced expression of *MCP-1* (Figure 4A). Contrarily, though unexpected, the pre-stimulation with *L. mucosae* CRL2069 for 24 and 48 h resulted in upregulation of LPS-induced *MCP-1* expression in BME cells, similarly to Pam3CSK4 (Figure 4A). The expression of *MCP-1* in *L. fermentum* CRL2085-treated BME cells was also significantly lower than control cells after 24 h of pre-stimulation.

The chemokine (C-X-C motif) ligand 3 (*CXCL3*) or macrophage inflammatory protein 2 beta (MIP-2 *β*) controls the migration and adhesion of monocytes. The pre-stimulation with the four *L. mucosae* CRL2069, *L. acidophilus* CRL2074, *L. rhamnosus* CRL2084, and *L. fermentum* CRL2085 were able to reduce the LPS-induced expression of *CXCL3* in BME cells (Figure 4B). Results indicated that CRL2074 and CRL2084 showed the most significant effects, and 12 h pre-stimulation showed stronger influence on immunomodulatory activities of LAB strains against LPS-induced inflammation (Figure 4B). However, 48 h of Pam3CSK4 pre-stimulation resulted in upregulation of the LPS-induced *CXCL3* expression in BME cells (Figure 4B).

### 2.5. Modulation of Negative Regulators of LPS-Induced Inflammation in BME Cells by Lactobacilli 

**Figure 5:**
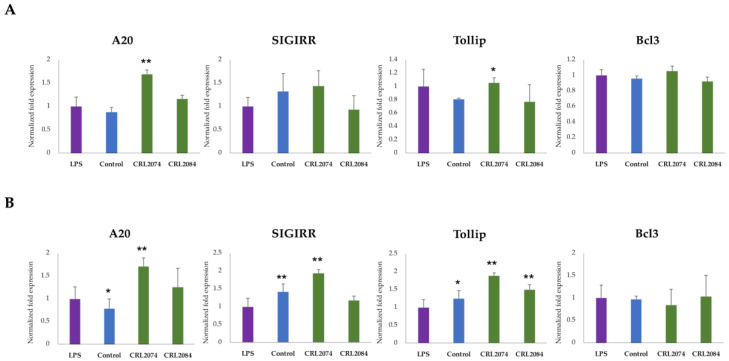
Evaluation of the immunomodulatory activity of lactobacilli in bovine mammary epithelial (BME) cells. BME cells (2.5 × 10^5^ cells/well) were pre-stimulated either with *L. acidophilus* CRL2074 or *L. rhamnosus* CRL2084 for 12 h, followed by challenge with lipopolysaccharides (LPS) for further 3 h (**A**) or 6 h (**B**). Then, mRNA expression of A20, single immunoglobulin IL-1 single receptor (SIGIRR), Toll interacting protein (Tollip), and B cell lymphoma 3 (Bcl3) were measured by RT-qPCR. Results presented are as mean ± SD of three independent experiments. LPS, LPS-challenged cells without LAB pre-stimulation; Control, untreated control cells; * *p* < 0.05, ** *p* < 0.01, *** *p* < 0.001 are the significant differences against LPS.

Because two strains, *L. acidophilus* CRL2074 and *L. rhamnosus* CRL2084, exerted anti-inflammatory properties in LPS-challenged BME cells having a 12 h pre-stimulation, we further tested whether they were able to upregulate the expression of negative regulators of the TLR4 signaling pathway. The pre-stimulation of both *L. acidophilus* CRL2074 and *L. rhamnosus* CRL2084 resulted in increased expression of the negative regulators ubiquitin editing enzyme A20 (also called TNF alpha-induced protein 3 or TNFAIP3), single immunoglobulin IL-1 single receptor (SIGIRR), and Toll interacting protein (Tollip) in MBE cells (Figure 5). The expression of B cell lymphoma 3 (Bcl3) in LPS-challenged BME cells remained stable irrespective of LAB pre-stimulation (Figure 5A,B). The pre-stimulation of CRL2074 strain resulted in significant upregulation A20 Tollip at 3 h post-LPS challenge to BME cells (Figure 5A). Notably, the expressions of A20, SIGIRR, and Tollip were significantly upregulated in CRL2074 pretreated BME cells when they were challenged with LPS for 6 h (Figure 5B). The CRL2084 pre-stimulation resulted in no significant changes in expression of any of four factors tested after 3 h LPS challenge (Figure 5A), but resulted in an increased expression of Tollip after 6 h of LPS challenge (Figure 5B). 

## 3. Discussion

The current mastitis control strategies in dairy farms largely involve either local intramammary infusion or parenteral administration of antibiotics. Indiscriminate antibiotic therapy to the lactating cow not only poses the risk of residual effect to humans through contaminated milk consumption, but also reduces the antibiotic efficacy due to emergence of antimicrobial-resistant mastitis pathogens [29]. The legal restrictions for using antibiotics in several developed countries as well as the continuous emergence of global antimicrobial resistant pathogens make imperative the exploration of effective substitutes for antibiotics. Several alternative approaches including the application of immunomodulatory beneficial microbes are being explored for preventing mastitis in dairy cows. It is known that *Lactobacillus* strains are diverse in their immunoregulatory properties, and therefore those properties need to be rigorously evaluated before the application of these live microorganisms into the bovine mammary gland. Moreover, even though lactobacilli are well known beneficial bacterial species and several strains are able to exert positive effects against subclinical bovine mastitis [20,21], it was recently reported in a murine model that the intramammary infusion of certain lactobacilli strains can be the cause of mastitis [19]. In this work, we presented a bovine mammary epithelial cells in vitro system that can be valuable for the selection and characterization of immunobiotic LAB intended for the prevention of mastitis. In addition, the present study evaluated the immunomodulatory potential of LAB strains isolated from dairy cattle environments on bovine mammary epithelial cells, and selected candidates that can be further studied in vivo.

The use of primary cells for in vitro investigation has the advantages of reflecting appropriate mitogenic responses as well as preserved physiological functions, including those related to the generation of immune responses. However, the isolation of epithelial cells from mammary gland tissue is costly, difficult, and allows only single and short-term experiments [30]. On the contrary, cell lines have advantages over primary cultures in that they are able to replicate for numerous passages, retaining reasonably constant cellular characteristics. The BME cell line used in the present study was cloned from the mammary tissue of a 200-day pregnant Holstein cow, and it was demonstrated that this cell line was able to respond to mitogenic stimulations [24]. Moreover, we recently reported that the BME cell line has an epithelial-like morphology, expresses members of the TLR family, and is able to induce proinflammatory cytokine and chemokine responses after the stimulation with LPS or *S. aureus* [25]. In particular, LPS stimulation significantly upregulated the expression of cytokines (*IL-1α* and *IL-1β* ) and chemokines (*IL-8, MCP-1, CXCL2*, and *CXCL3*) in BME cells [25]. In a similar approach, we have also demonstrated that the BME cell line is a useful tool for investigating the molecular interaction between host epithelial cells and pathogenic or beneficial microorganisms, in particular the mechanisms through which microorganisms modulate the host epithelial immune responses [31,32]. We therefore postulated that BME cells have the potential to be used in a similar way in order to evaluate the cellular and molecular interactions of microorganisms with bovine epithelial cells in the mammary gland, thereby providing an efficient in vitro selection tool for immunobiotic candidates against bovine mastitis.

The experiments carried out here were based on the hypothesis that feedlot cattle LAB strains are able to induce a certain degree of innate immune modulation in BME cells through their interaction with TLRs, and that the transcriptomic changes induced in the bovine cells by this interaction can inhibit or minimize the subsequent pathogen-induced inflammatory reaction. This approach has been useful for the selection of immunomodulatory LAB, as we have described previously for other porcine and bovine in vitro immunoassay systems [32,33,34]. Therefore, in the BME cells in vitro evaluation system, the cells were pre-stimulated with the different feedlot cattle LAB strains for a certain period and then challenged with a pathogen-derived bioactive molecule. It was reported that the duration of the bacterial pre-stimulation significantly affects the epithelial cellular immune responses [31,32,34]. Therefore, we performed experiments with three different periods (12, 24, and 48 h) of LAB pre-stimulations in order to evaluate the temporal effects of LAB pretreatment in the modulation of the subsequent inflammatory challenge. On the other hand, we selected LPS as the inflammatory challenge, taking into consideration that *E. coli* is one of the major mastitis-causing pathogens that induces acute inflammation of the mammary gland through the activation of the LPS/TLR4-mediated signaling pathway [4,5]. 

In our study, the most remarkable effects on the expressions of inflammatory factors were found when a period of 12 h was used to stimulate BME cells with feedlot cattle LAB strains, before the challenge with LPS. The expression of *IL-1α*, *IL-1β*, *MCP-1, CXCL2*, and *CXCL3* in BME cells were differentially modulated by at least one of the LAB strains after 12 h of pre-stimulation. On the other hand, both 12 and 24 h pre-stimulations markedly modulated *IL-8* expression. These results indicate that short contact times with LAB would be more efficient to achieve optimal immunomodulatory effects on the mammary gland epithelium in the context of TLR4-mediatted inflammation. This finding contrasts with our previous results obtained in other porcine and bovine in vitro immunoassay systems. When immunomodulatory LAB strains were searched in BIE cells or porcine intestinal epithelial (PIE) cells, it was found that among the several pre-stimulation times evaluated, 48 h was the most appropriate time to obtain optimal modulation of epithelial inflammatory responses [31,33,34]. Some other related studies have also reported that prolonged stimulation times correlated with optimal immunomodulatory capacities for LAB strains in intestinal systems [35,36]. These differences found between the intestinal and the mammary gland in vitro systems may be related to the physiology of each mucosal tissue from which the epithelial cells derive. Intestinal epithelial cells, more adapted to being in contact with microorganisms, would require a longer period of contact to respond with transcriptomic changes that modify innate immune responses. On the other hand, mammary gland epithelial cells would be less adapted to be contact with microorganisms and therefore they would have a decreased tolerance to microbial molecules. These results presuppose practical limitations in the use of LAB in vivo in the bovine host, as it would be necessary to achieve a short stimulation time. However, it must be kept in mind that the epithelial cells of the mammary gland are immersed in a complex microenvironment and are influenced by various factors such as hormones, immune and non-immune cells, as well as microorganisms from the normal microbiota. In these circumstances, the immunomodulatory capacity of LABs could be modified and a longer stimulation period could be necessary. Undoubtedly, in vivo kinetic studies would be of great value to optimize LAB treatments aimed at achieving optimal beneficial immunomodulatory effects in the bovine mammary gland.

**Figure 6:**
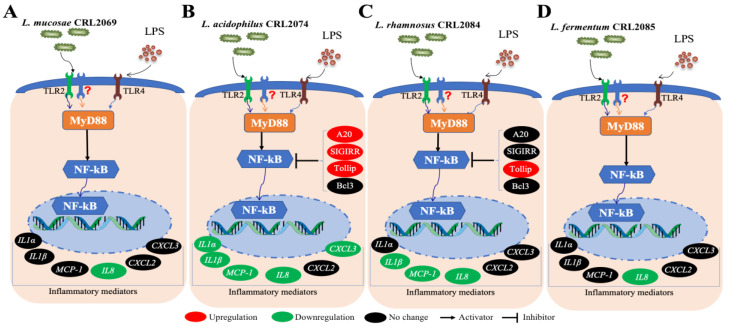
Proposed mechanism of LAB-mediated modulation of inflammatory responses in bovine mammary epithelial (BME) cells. Being Gram-positive bacteria, immunobiotic strains when pre-stimulated to BME cells were recognized by TLR2 and induced a downstream signaling cascade of NFkB transcription activation through adaptor protein MyD88, initiating the secretion of cytokine and chemokine profiles. The LPS derived from *Escherichia coli*, when used to challenge the immunobiotic-sensitized BME cells, was recognized by TLR4 and followed the same pathway for NFkB activation through MyD88, inducing inflammatory responses. Thus, having a strain-dependent variation, immunobiotic-mediated pre-activation of TLR2 might influence the subsequent TLR4-induced inflammation. *L. acidophilus* CRL2074 and *L. rhamnosus* CRL2084 showed the highest ability to differentially modulate the expression of proinflammatory cytokines and chemokines in LPS-challenged BME cells. In addition, several negative regulators of TLR signaling also influenced the resultant cytokine and chemokine profiles. The *L. acidophilus* CRL2074 pre-exposure was able to upregulate A20, SIGIRR, and Tollip expression, whereas *L. rhamnosus* CRL2084 only upregulated the expression of Tollip.

As demonstrated for almost all probiotic properties, the ability of LAB to modulate the TLR4-mediated immune response in BME cells was a strain-dependent characteristic (Figure 6). Among the feedlot cattle lactobacilli evaluated in this work, *L. acidophilus* CRL2074 and *L. rhamnosus* CRL2084 showed the highest ability to differentially modulate the expression of proinflammatory cytokines and chemokines in LPS-challenged BME cells. *L. acidophilus* CRL2074 was able to reduce the expression of all the inflammatory factors evaluated with the exception of *CXCL2*, whereas *L. rhamnosus* CRL2084 reduced *IL-1β*, *MCP-1*, and *IL-8*. 

In intramammary gland infections, the invading bacteria start to multiply within the alveolus and liberate toxins that induce the release of inflammatory factors by epithelial cells and resident leukocytes, which attracts blood neutrophils and monocytes to the site of infection [37,38]. The recruitment and activation of immune cells is a hallmark response in the host–pathogen interaction during intramammary gland infections. A large influx of neutrophils occurs after the infectious challenge, and the concentration of those phagocytic cells in the infected tissue correspond to the inflammatory response intensity [4,39,40]. Although the phagocytic and microbicidal activity of neutrophils is important for limiting the infection and eliminating the pathogen, if this response is not properly regulated it can lead to local tissue damage, contributing to worsening the course of the disease [37,41]. Oxidant compounds and enzymes released by neutrophils result in mammary alveolar atrophy, epithelial damage, and breaching of the blood barrier, increasing the susceptibility to systemic infection [37,38]. Thus, uncontrolled inflammation in the mammary gland can be harmful, even fatal, for the bovine host [42,43].

The modulation of cytokine and chemokine expression may offer novel approaches in the prevention or treatment of bovine mastitis [41]. In this regard, *L. acidophilus* CRL2074 significantly reduced the expression of *IL-1α, IL-8, MCP-1*, and *CXCL3* in LPS-challenged BME cells. Pro-inflammatory cytokine *IL-1α* is important to elicit an acute phase response of intramammary inflammation [38]. On the other hand, chemokines such as *IL-8* attract polymorphonuclear leukocytes from the blood to the infection site, which is reflected by an increase of somatic cell count (SSC) in milk [44]. The immediate recruitment of somatic cells from the blood into the udder is essential for effective elimination of intramammary pathogens [38]. *MCP-1* and *CXCL3* attract mononuclear leukocytes such as monocytes, natural killer cells, and activated lymphocytes [45]. It was demonstrated that MCP-1 induces cell proliferation through activation of phosphatidylinositol 3 kinase (PI3K)/protein kinase B (PKB, also called AKT) and mitogen-activated protein kinase (MAPK) pathways, and prevents the LPS-induced inflammatory responses in bovine mammary epithelial cells (MAC-T cell line) [46]. Thus, immunobiotic *L. acidophilus* CRL2074 capable of reducing the magnitude of leukocyte migration into the udder, which is reflected in the expression of *IL-1α, IL-8, MCP-1,* and *CXCL3*, might influence the clinical course of mastitis.

In the present study, *L. acidophilus* CRL2074 pre-stimulated BME cells showed significant upregulation of A20, SIGIRR, and Tollip expression after LPS challenge. The ubiquitin-editing enzyme A20 is recognized as a key regulator of TLR4 signaling, particularly involved in the negative feedback regulation of NF-kB activation in the intestinal epithelial cells [47,48]. To the best of our knowledge, the effect of A20 and SIGIRR in the regulation of inflammatory responses in bovine mammary epithelium has not been reported before. Thus, our results would indicate for the first time the role of A20 and SIGIRR in the control of bovine mammary gland TLR4-triggered inflammation. On the other hand, Tollip has been implicated in the negative regulation of LPS-induced TLR4 signaling through the modulation of proinflammatory cytokines in bovine mammary epithelial cells [49]. Thus, the ability of the CRL2074 strain to differentially modulate the expression of three negative regulators when compared with the CRL2084 strain that regulated only a single factor would explain the higher ability of *L. acidophilus* CRL2074 to diminish proinflammatory factors expressions in the bovine mammary gland (Figure 6).

In conclusion, our results demonstrated that the *L. acidophilus* CRL2074 strain possesses remarkable immunomodulatory abilities against LPS-induced inflammation in BME cells. This *Lactobacillus* strain could be used as candidate for in vivo testing their beneficial effects in bovine mastitis through intramammary infusion. Our findings also suggest that the BME cells immunoassay system could be of value for the in vitro evaluation of the immunomodulatory abilities of LAB against the inflammation resulted from the intramammary infection with mastitis-related pathogens. 

## 4. Materials and Methods 

### 4.1. Cell Line and Culture Condition

The BME cell line used in this work was originally established by our group [25]. BME cells were derived from the mammary tissue taken from a 200 day pregnant Holstein Frisian cow. BME cells were maintained in Dulbecco’s modified Eagle’s medium (DMEM; Gibco Paisley, Scotland, United Kingdom) supplemented with 20% fetal calf serum (FCS; Sigma-Aldrich, Tokyo, Japan), 100 U/mL penicillin and 100 μg/mL streptomycin (Gibco 15140122, Life Science Technologies), transferrin (5 mg/mL), and sodium acetate (5mM) as growth medium. For the passage, the BME cells were plated at the concentration of 2.5 × 10^5^ cells/cm^2^ in the 6-well cell culture plates (BD Falcon, Tokyo, Japan) and incubated in a humidified atmosphere of 5% CO_2_. The cell culture medium was changed every 24 h. The cells used in this study belong to 22th to 28th passages. 

### 4.2. Growth and Maintenance of Microorganisms

Four lactobacillus strains used in this study were obtained from CERELA-CONICET (Tucuman, Argentina). The LAB strains (Table 1) were originally isolated from the feedlot cattle’s environment [26].

LAB strains were grown in Man, Rogosa, and Sharpe broth (MRS, Britania) at 37 °C for 18 h, and kept in milk yeast extract (10 g low fat milk, 0.5 h yeast extract, and 1 g glucose per 100 mL) with 12% glycerol at −20 °C. Before performing the experimental assays, bacteria were subcultured three times every 12–14 h at 37 °C in MRS broth. For preparing the bacterial inoculum, a culture of the strain (10^9^ CFU) after incubation for 48 h at 37 °C in MRS broth was centrifuged, and the bacterial pellete was washed twice with saline solution (0.8% NaCl). Cells were suspended in 5 mL of DMEM (10% FCS, 1% SP) and counted under a microscope using a Petroff–Hausser counting chamber. The cell suspension was adjusted to a concentration of 10^9^ CFU/mL followed by serial dilution in saline solution to have a concentration of 10^6^ CFU/mL. This inoculum was fractionated and stored at 4 °C until the experiment was performed within a period no longer than 2 h [50]. 

### 4.3. Immunobiotic Evaluation Assay in BME Cells 

In a first set of experiments, BME cells (5 × 10^5^ cells per well) were plated in a 12-well cell culture plate and stimulated with LPS (1.0 μg/mL) or Pam3CSK4 (200 ng/mL). The cultures were maintained at 37 °C in a humidified atmosphere of 5% CO_2_ for 3, 6, 12, or 24 h, and then the treated cells were harvested along with untreated control cells for measuring the mRNA expression of TLRs.

In second set of experiments, BME cells were seeded on a 24-well plate at 1.25 × 10^5^ cells per well (500 μL) and cultured for 2 days at 37 °C in CO_2_. When reached monolayer confluence on the third day of culture (2.5 × 10^5^ cells per well), suspension of *L. mucosae* CRL2069, *L. acidophilus* CRL2074, *L. rhamnosus* CRL2084, or *L. fermentum* CRL2085 (5.0 × 10^7^ cells per well) or Pam3CSK4 (200 ng/mL) were added to the BME cultures. The LAB strains were subjected to heat treatment at 72 °C for 90 min prior to stimulation. The bacterial stimulation of BME cell culture was performed for 12, 24, or 48 h at 37 °C in 5% CO_2_. At each time point, LPS (1.0 μg/mL) was added after washing the cells twice with PBS. BME cells were further cultured for 3, 6, or 12 h at 37 °C in 5% CO_2_ and cells were harvested for RNA isolation at each time point.

### 4.4. Real-Time Quantitative PCR

Two-step real-time PCR (RT-qPCR) was performed for quantification of selected mRNAs in BME cells. Total RNA was extracted from the treated and untreated BME cells using PureLink RNA Mini kit (Life Technologies Inc., Carlsbad, USA) along with on-column DNase treatment. The primer sequences used are presented in Table 2. The cDNA synthesis was performed using the quantiTect reverse transcription kit (Qiagen, Tokyo, Japan) according to the manufacturer’s recommendations. RT-qPCR was performed using a 7300 real time PCR system (Applied Biosystem, Warrington, United Kingdom) using the TaqMan gene expression assay kit (Life Technologies Inc., Carlsbad, USA) and TaqMan Universal Master Mix II with UNG (Applied Biosystem, Warrington, United Kingdom). The PCR thermal cycling conditions were 2 min at 50 °C, followed by 10 min at 95 °C, and then 40 cycles of 15 s at 95 °C, 1 min at 60 °C. The reaction mixtures contained 2.5 μL of cDNA, 1 μL of gene expression assay, 10 μL of TaqMan Universal Mix II with UNG, and 6.5 μL distilled water. According to the minimum information for publication of RT-qPCR experiments guidelines, β-actin was used as a housekeeping gene because of its high stability across various bovine tissue [51]. Relative index was calculated as the ratio of target gene expression of β-actin. Then, raw data were transformed from the mean CT values of replicated samples to copy number of the established standard curve.

### 4.5. Statistical Analysis 

The raw data were log_2_ transformed followed by a normality check using the Kolmogrov–Smirnov test. Comparisons between mean values were carried out using one-way ANOVA and Fisher’s least significant different test. For every case, *p* < 0.05 was considered statistically significant. 

## 5. Conclusions

Taking into consideration that the appropriate regulation of the production of proinflammatory mediators is highly important to maintain immune homeostasis and to prevent excessive inflammatory injury to the mammary gland, *L. acidophilus* CRL2074 may have the potential to be used as an immunobiotic strain to prevent and to treat bovine intramammary infections. This *Lactobacillus* strain could be used as a candidate for in vivo testing of beneficial effects in bovine mastitis through intramammary infusion. Our findings also suggest that the BME cells immunoassay system could be a useful tool for the evaluation of the immunomodulatory abilities of LAB against the inflammation resulting from the intramammary infection with mastitis-related pathogens. 

## Figures and Tables

**Table 1:** Lactic acid bacteria used in this study.

Species	Strain ID	Source of Origin
*Lactobacillus mucosae*	CRL2069	cattle feces/pen soil/feed rations
*Lactobacillus acidophilus*	CRL2074	cattle feces/pen soil/feed rations
*Lactobacillus rhamnosus*	CRL2084	cattle feed rations
*Lactobacillus fermentum*	CRL2085	cattle feed rations

**Table 2:** Sequence of the primer sets used for gene expression study by RT-qPCR.

Gene Symbol	Sequence (3′ to 5′) *	Amplicon Size	Accession Number
*ACTB*	F: TGG ATT GGC GGC TCC AT R: GCT GAT CCA CAT CTG CTG GAA	57	NM_173979.3
*IL-1* *α*	F: CAG TTG CCC ATC CAA AGT TGT T R: TGC CAT GTG CAC CAA TTT TT	59	NM_174092.1
*IL-1* *β*	F: GAG CCT GTC ATC TTC GAA ACG R: GCA CGG GTG CGT CAC A	55	NM_174093.1
*CCL2*	F: CAC CAG CAG CAA GTG TCC TAA A R: CAC ATA ACT CCT TGC CCA GGA T	65	NM_174006.2
*CXCL2*	F: CTA GGC CAG CTC TAA CTG AC R: TGG TGA TTC CTC TTT TCC CT	107	NM_174299.3
*CXCL3*	F: GAC AGT TCC TGA AAA GTG GT R: ATA GTC CAG CAC ATC AAG TC	104	NM_001046513.2
*IL-8*	F: TGC TCT CTT GGC AGC TTT CC R: TCT TGA CAG AAC TGC AGC TTC AC	61	NM_173925.2
*TLR2*	F: GGG TGC TGT GTC ACC GTT TC R: GCC ACG CCC ACA TCA TCT	57	NM_174197.2
*TLR4*	F: AGC ACC TAT GAT GCC TTT GTC A R: GTT CAT TCC GCA CCC AGT CT	61	NM_174198.6
*A20*	F: AAAGTGGGCTGCATGTACTTTGG R: AGGCTGTGGGACTGGCTTTC	121	NM_001192170.1
*SIGIRR*	F: GGCAGTGAAGTGGATGTGTCA R: TCCGTGCGGGCACTGTA	56	NM_001082443
*Tollip*	F: CGGGCGTGGACTCTTTCTAC R: GATGCGGTCGTCCATGGA	65	NM_001039961
*Bcl3*	F: CATGGAACACCCCCTGTCA R: GGCGTATCTCCATCCTCATCA	66	NM_001205993

* F, Forward; R, Reverse.

## References

[1] Halasa T., Huijps K., Østerås O., Hogeveen H. (2007). Economic effects of bovine mastitis and mastitis management: A review. Vet. Q..

[2] Jensen K., Günther J., Talbot R., Petzl W., Zerbe H., Schuberth H.J., Seyfert H.M., Glass E.J. (2013). Escherichia coli- and Staphylococcus aureus-induced mastitis differentially modulate transcriptional responses in neighbouring uninfected bovine mammary gland quarters. BMC Genom..

[3] Schukken Y.H., Günther J., Fitzpatrick J., Fontaine M.C., Goetze L., Holst O., Leigh J., Petzl W., Schuberth H.-J., Sipka A. (2011). Host-response patterns of intramammary infections in dairy cows. Vet. Immunol. Immunopathol..

[4] Burvenich C., Van Merrid V., Mehrzad J., ez-Fraile A., Duchateau L. (2003). Severity of *E. coli* mastitis is mainly determined by cow factors. Vet. Res..

[5] Mitterhuemer S., Petzl W., Krebs S., Mehne D., Klanner A., Wolf E., Zerbe H., Blum H. (2010). Escherichia coli infection induces distinct local and systemic transcriptome responses in the mammary gland. BMC Genom..

[6] Taponen S., Jantunen A., Pyoerala E., Pyoerala S. (2003). Efficacy of targeted 5 day combined parenteral and intramammary treatment of clinical mastitis caused by penicillin-susceptible or penicillin-resistant Staphylococcus aureus. Acta Vet. Scand..

[7] Bradley A.J., Breen J.E., Payne B., Williams P., Green M.J. (2010). The use of a cephalonium containing dry cow therapy and an internal teat sealant, both alone and in combination. J. Dairy Sci..

[8] Bradley A.J., Breen J.E., Payne B., Green M.J. (2011). A comparison of broad-spectrum and narrow-spectrum dry cow therapy used alone and in combination with a teat sealant. J. Dairy Sci..

[9] Dalton J.C. (2006). Antibiotic residue prevention in milk and dairy beef. West. Dairy News.

[10] Saini V., McClure J.T., Léger D., Keefe G.C., Scholl D.T., Morck D.W., Barkema H.W. (2012). Antimicrobial resistance profiles of common mastitis pathogens on Canadian dairy farms. J. Dairy Sci..

[11] Cao L.T., Wu J.Q., Xie F., Hu S.H., Mo Y. (2007). Efficacy of Nisin in treatment of clinical mastitis in lactating dairy cows. J. Dairy Sci..

[12] FAO (2002). WHO 2002 Guidelines for the Evaluation of Probiotics in Food.

[13] Villena J., Aso H., Rutten V.P.M.G., Takahashi H., van Eden W., Kitazawa H. (2018). Immunobiotics for the Bovine Host: Their Interaction with Intestinal Epithelial Cells and Their Effect on Antiviral Immunity. Front. Immunol..

[14] Uyeno Y., Shigemori S., Shimosato T. (2015). Effect of Probiotics/Prebiotics on Cattle Health and Productivity. Microbes Environ..

[15] Chaimanee V., Sakulsingharoj C., Deejing S., Seetakoses P., Niamsup P. (2009). Screening and characterization of bacteriocin-producing bacteria capable of inhibiting the growth of bovine mastitis. Maejo Int. J. Sci. Technol..

[16] Espeche M.C., Otero M.C., Sesma F., Nader-Macias M.E.F. (2009). Screening of surface properties and antagonistic substances production by lactic acid bacteria isolated from the mammary gland of healthy and mastitic cows. Vet. Microbiol..

[17] Crispie F., Alonso-Gomez M., O’Loughlin C., Klostermann K., Flynn J., Arkins S., Meaney W., Ross R.P., Hill C. (2008). Intramammary infusion of a live culture for treatment of bovine mastitis: Effect of live lactococci on the mammary immune response. J. Dairy Res..

[18] Beecher C., Daly M., Berry D.P., Klostermann K., Flynn J., Meaney W., Hill C., McCarthy T.V., Ross R.P., Giblin L. (2009). Administration of a live culture of Lactococcus lactis DPC 3147 into the bovine mammary gland stimulates the local host immune response, particularly IL-1β and IL-8 gene expression. J. Dairy Res..

[19] Camperio C., Armas F., Biasibetti E., Frassanito P., Giovannelli C., Spuria L., D’Agostino C., Tait S., Capucchio M.T., Marianelli C. (2017). A mouse mastitis model to study the effects of the intramammary infusion of a food-grade Lactococcus lactis strain. PLoS ONE.

[20] Armas F., Camperio C., Marianelli C. (2017). In Vitro Assessment of the Probiotic Potential of Lactococcus lactis LMG 7930 against Ruminant Mastitis-Causing Pathogens. PLoS ONE..

[21] Klostermann K., Crispie F., Flynn J., Ross R.P., Hill C., Meaney W.J. (2008). Intramammary infusion of a live culture of Lactococcus lactis for treatment of bovine mastitis: Comparison of antibiotic treatment in field trials. J. Dairy Res..

[22] Pellegrino M., Berardo N., Giraudo J., Nader-Macias M.E.F., Bogni C. (2017). Bovine mastitis prevention: Humoral and cellular response of dairy cows inoculated with lactic acid bacteria at the dry-off period. Benef Microbes.

[23] Diepers A., Krömker V., Zinke C., Wente N., Pan L., Paulsen K., Paduch J.-H. (2017). In vitro ability of lactic acid bacteria to inhibit mastitis-causing pathogens. Sustain. Chem. Pharm..

[24] Rose T.M., Aso H., Yonekura S., Komatsu T., Hagino A., Ozutsumi K., Obara Y. (2002). In Vitro differentiation of a cloned bovine mammary epithelial cell. J. Dairy Res..

[25] Islam M.A., Takagi M., Fukuyama K., Komatsu R., Albarracin L., Nochi T., Suda Y., Ikeda-Ohtsubo W., Rutten V., Eden W. (2020). Transcriptome Analysis of The Inflammatory Responses of Bovine Mammary Epithelial Cells: Exploring Immunomodulatory Target Genes for Bovine Mastitis. Pathogens.

[26] Maldonado N.C., Ficoseco C.A., Mansilla F.A., Melian C., Hebert E.M., Vignolo G.M., Nader-Macias M.E.F. (2018). Identification, characterization and selection of autochthonous lactic acid bacteria as probiotic for feedlot cattle. Livest. Sci..

[27] Aristimuño F.C., Mansilla F.I., Maldonado N.C., Miranda H., Nader-Macias M.E.F., Vignolo G.M. (2018). Safety and Growth Optimization of Lactic Acid Bacteria Isolated from Feedlot Cattle for Probiotic Formula Design. Front. Microbiol..

[28] Mansilla F., Takagi M., Garcia-Castillo V., Aso H., Nader-Macias M.E., Vignolo G., Kitazawa H., Villena J. (2020). Modulation of Toll-like receptor-mediated innate immunity in bovine intestinal epithelial cells by lactic acid bacteria isolated from feedlot cattle. Benef. Microbes.

[29] Oliver S.P., Murinda S.E. (2012). Antimicrobial resistance of mastitis pathogens. Vet. Clin. N. Am. Food Anim. Pract..

[30] McGrath M.F. (1987). A novel system for mammary epithelial cell culture. J. Dairy Sci..

[31] Takanashi N., Tomosada Y., Villena J., Murata K., Takahashi T., Chiba E., Tohno M., Shimazu T., Aso H., Suda Y. (2013). Advanced application of bovine intestinal epithelial cell line for evaluating regulatory effect of lactobacilli against heat-killed enterotoxigenic Escherichia coli-mediated inflammation. BMC Microbiol..

[32] Kobayashi H., Kanmani P., Ishizuka T., Miyazaki A., Soma J., Albarracin L., Suda Y., Nochi T., Aso H., Iwabuchi N. (2017). Development of an in vitro immunobiotic evaluation system against rotavirus infection in bovine intestinal epitheliocytes. Benef. Microbes.

[33] Garcia-Castillo V., Albarracin L., Kitazawa H., Villena J., Kanauchi M. (2019). Screening and characterization of immunobiotic Lactic Acid bacteria with porcine immunoassay systems. Lactic Acid Bacteria. Methods in Molecular Biology.

[34] Miyazawa K., Hondo T., Kanaya T., Tanaka S., Takakura I., Itani W., Rose M.T., Kitazawa H., Yamaguchi T., Aso H. (2010). Characterization of newly established bovine intestinal epithelial cell line. Histochem. Cell Biol..

[35] Wu Q., Lie M.-C., Yang J., Wang J.-F., Zhu W.-H. (2016). Lactobacillus rhamnosus GR-1 Ameliorates Escherichia coli-Induced Inflammation and Cell Damage via Attenuation of ASC-Independent NLRP3 Inflammasome Activation. Appl. Environ. Microbiol..

[36] Taranu I., Martin D.E., Braicu C., Pistol G.C., Sorescu I., Pruteanu L.L., Neagoe I.B., Vodnar D.C. (2018). In Vitro Transcriptome Response to a Mixture of Lactobacilli Strains in Intestinal Porcine Epithelial Cell Line. Int. J. Mol. Sci..

[37] Viguier C., Arora S., Gilmartin N., Welbeck K., O’Kennedy R. (2009). Mastitis detection: Current trends and future perspectives. Trends Biotechnol..

[38] Rainard P., Riollet C. (2006). Innate immunity of the bovine mammary gland. Vet. Res..

[39] Kopp E., Medzhitov R. (2003). Recognition of microbial infection by Toll-like receptors. Curr. Opin. Immunol..

[40] Paape M., Mehrzad J., Zhao X., Detilleux J., Burvenich C. (2002). Defense of the bovine mammary gland by polymorphonuclear neutrophil leukocytes. J. Mammary Gland Biol..

[41] De Galdeano C.M., LeBlanc A.d.M., Vinderola G., Bonet M.E.B., Perdigon G. (2007). Minireview. Proposed model: Mechanisms of immunomodulation induced by probiotic bacteria. Clin. Vaccine Immunol..

[42] Liew F.Y., Xu D., Brint E.K., O’Neill L.A. (2005). Negative regulation of toll-like receptor-mediated immune responses. Nat. Rev. Immunol..

[43] Liu M., Song S., Li H., Jiang X., Yin P., Wan C., Liu X., Liu F., Xu J. (2014). The protective effect of caffeic acid against inflammation injury of primary bovine mammary epithelial cells induced by lipopolysaccharide. J. Dairy Sci..

[44] Oviedo-Boyso J., Valdez-Alarcón J.J., Cajero-Juárez M., Ochoa-Zarzosa A., López-Meza J.E., Bravo-Pantino A., Baizabal-Aguirre V.M. (2007). Innate immune response of bovine mammary gland to pathogenic bacteria responsible for mastitis. J. Infect..

[45] Lee P.Y., Li Y., Kumagai Y., Xu Y., Weinstein J.S., Kellner E.S., Nacionales D.C., Butfiloski E.J., van-Rooijen N., Akira S. (2009). Type I interferon modulates monocyte recruitment and maturation in chronic inflammation. Am. J. Pathol..

[46] Yang C., Lim W., Bae H., Bazer F.W., Song G. (2018). C-C motif chemokine ligand 2 induces proliferation and prevents lipopolysaccharide-induced inflammatory responses in bovine mammary epithelial cells. J. Dairy Sci..

[47] Vereecke L., Sze M., Mc Guire C., Rogiers B., Chu Y., Schmidt-Supprian M., Pasparakis M., Beyaert R., van Loo G. (2010). Enterocyte-specific A20 deficiency sensitizes to tumor necrosis factor-induced toxicity and experimental colitis. J. Exp. Med..

[48] Tomosada Y., Villena J., Murata K., Chiba E., Shimazu T., Aso H., Iwabuchi N., Xiao J.-X., Saito T., Kitazawa H. (2013). Immunoregulatory Effect of Bifidobacteria Strains in Porcine Intestinal Epithelial Cells through Modulation of Ubiquitin-Editing Enzyme A20 Expression. PLoS ONE.

[49] Ibeagha-Awemu E.M., Lee J.W., Ibeagha A.E., Bannerman D.D., Paape M.J., Zhao X. (2008). Bacterial lipopolysaccharide induces increased expression of toll-like receptor (TLR) 4 and downstream TLR signaling molecules in bovine mammary epithelial cells. Vet. Res..

[50] Frola I.D., Pellegrino M.S., Espeche M.C., Giraudo J.A., Nader-Macias M.E.F., Bogni C.I. (2011). Effects of intramammary inoculation of Lactobacillus perolens CRL1724 in lactating cows udders. J. Dairy Res..

[51] Bustin S.A., Benes V., Garson J.A., Hellemans J., Huggett J., Kubista M., Mueller R., Nolan T., Pfaffl M.W., Shipley G.L. (2009). The MIQE guidelines: Minimum information for publication of quantitative real-time PCR experiments. Clin. Chem..

