# Erratum: Fukuyama, K., et al. Evaluation of the Immunomodulatory Ability of Lactic Acid Bacteria Isolated from Feedlot Cattle Against Mastitis Using a Bovine Mammary Epithelial Cells In Vitro Assay. *Pathogens* 2020, *9*, 410

**DOI:** 10.3390/pathogens9070574

**Published:** 2020-07-16

**Authors:** Kohtaro Fukuyama, Md. Aminul Islam, Michihiro Takagi, Wakako Ikeda-Ohtsubo, Shoichiro Kurata, Hisashi Aso, Maria Elena Fatima Nader-Macías, Graciela Vignolo, Julio Villena, Haruki Kitazawa

**Affiliations:** 1 Food and Feed Immunology Group, Laboratory of Animal Products Chemistry, Graduate School of Agricultural Science, Tohoku University, Sendai 980-8572, Japan; fukuyama.k.mc0511@gmail.com (K.F.); aminul.vmed@bau.edu.bd (M.A.I.); takagimichihiro@gmail.com (M.T.); wakako.ohtsubo.a7@tohoku.ac.jp (W.I.-O.); 2 Livestock Immunology Unit, International Education and Research Centre for Food and Agricultural Immunology (CFAI), Graduate School of Agricultural Science, Tohoku University, Sendai 980-8572, Japan; asosan@tohoku.ac.jp; 3 Department of Medicine, Faculty of Veterinary Science, Bangladesh Agricultural University, Mymensingh 2202, Bangladesh; 4 Laboratory of Molecular Genetics, Graduate School of Pharmaceutical Sciences, Tohoku University, Sendai 980-8572, Japan; shoichiro.kurata.d5@tohoku.ac.jp; 5 Cell Biology Laboratory, Graduate School of Agricultural Science, Tohoku University, Sendai 980-8572, Japan; 6 Reference Laboratory for Lactobacilli, (CERELA-CONICET), Tucuman-4000, Argentina; fnader@cerela.org.ar

The authors would like to make the following corrections about the published paper [1]. The changes are as follows:

(1) Adding the author name and email address:
  Maria Elena Fatima Nader-Macías;
  fnader@cerela.org.ar;
(2) Adding the author contributions of Maria Elena Fatima Nader-Macías in the Author Contributions section:
  resources; selection of strains.
(3) Adding the following sentence in the Funding section:
  This work was also supported by the grants: CONICET PIP 744; PDTS CONICET-TRIGOTUC S.A. 0289; MINCYT-ANPCYT PICT 1187; MINCYT-FONTAR-EMPRETECNO 002-2016.

The authors and the Editorial Office would like to apologize for any inconvenience caused to the readers by these changes. The change does not affect the scientific results. The manuscript will be updated and the original will remain online on the article webpage.
